# *Depreissia decipiens*, an enigmatic canopy spider from Borneo revisited (Araneae, Salticidae), with remarks on the distribution and diversity of canopy spiders in Sabah, Borneo

**DOI:** 10.3897/zookeys.556.6174

**Published:** 2016-01-21

**Authors:** Christa L. Deeleman-Reinhold, Jeremy Miller, Andreas Floren

**Affiliations:** 1 Sparrenlaan 8 4641GA Ossendrecht, The Netherlands; 2 Department of Terrestrial Zoology, Naturalis Biodiversity Centre, Postbus 9517, 233 3 CR Leiden, The Netherlands; 3 Department of Animal Ecology and Tropical Ecology, Biozentrum Am Hubland, Universität Würzburg, 97074 Würzburg, Germany

**Keywords:** Jumping spiders, canopy spiders, taxonomy, biodiversity, ant-mimicking spiders, wasp-mimicking, Mt. Kinabalu, rainforest, Cocalodinae, Polistine wasps, endemism

## Abstract

*Depreissia* is a little known genus comprising two hymenopteran-mimicking species, one found in Central Africa and one in the north of Borneo. The male of *Depreissia decipiens* is redescribed, the female is described for the first time. The carapace is elongated, dorsally flattened and rhombus-shaped, the rear of the thorax laterally depressed and transformed, with a pair of deep pits; the pedicel is almost as long as the abdomen. The male palp is unusual, characterized by the transverse deeply split membranous tegulum separating a ventral part which bears a sclerotized tegular apophysis and a large dagger-like retrodirected median apophysis. The female epigyne consists of one pair of large adjacent spermathecae and very long copulatory ducts arising posteriorly and rising laterally alongside the spermathecae continuing in several vertical and horizontal coils over the anterior surface. Relationships within the Salticidae are discussed and an affinity with the Cocalodinae is suggested. Arguments are provided for a hypothesis that *Depreissia decipiens* is not ant-mimicking as was previously believed, but is a mimic of polistinine wasps. The species was found in the canopy in the Kinabalu area only, in primary and old secondary rainforest at 200–700 m.a.s.l. Overlap of canopy-dwelling spider species with those in the understorey are discussed and examples of species richness and endemism in the canopy are highlighted. Canopy fogging is a very efficient method of collecting for most arthropods. The canopy fauna adds an extra dimension to the known biodiversity of the tropical rainforest. In southeast Asia, canopy research has been neglected, inhibiting evaluation of comparative results of this canopy project with that from other regions. More use of fogging as a collecting method would greatly improve insight into the actual species richness and species distribution in general.

## Introduction

Lessert (1942) first reported the male of a strange ant-mimicking salticid spider from central Africa for which he established the genus *Depreissia*. *Depreissia myrmex* Lessert, 1942 is a small (3.2 mm total length) dark spider with elongate carapace, flat and low, with palps structured basically different from those of other Salticidae. The female is unknown. Wesołowska (1997) redescribed the type specimen, adding more details of the palp. As to the affinities of this spider, she was unable to indicate any close relationships.

A second species from secondary rainforest in the Kinabalu region of Malaysian Borneo, described as *Depreissia decipiens* Deeleman-Reinhold & Floren, 2003, exhibits remarkably close similarity to the type species. Like its African relative, only the male was known at the time of description (Deeleman-Reinhold and Floren 2003). A female was discovered later in samples from a primary forest near the forest where the male was found; it is described here. How can this disjunct distribution be explained? Are they relict species, only survivors of an ancient fauna, are they linked by intermediate unknown populations and species?

In this paper, the genital organs of male and female are (re)described and analysed. Phylogenetic affinities are suggested based on the structure of the male and female genitalia. Like the genus *Athamas* O. Pickard-Cambridge and the lyssomanine salticids, the eyes of *Depreissia myrmex* are arranged in four rows (this is not so in *Depreissia decipiens*). These groups otherwise seem phylogenetically remote. Only two adult specimens and four immatures of *Depreissia* were found amongst an estimated 15,000 spiders that were extracted from an ecological project of canopy fogging arthropods in a variety of forest types and localities through Sabah, Borneo. Possible models of mimicry are discussed.

## Methods

*Depreissia* specimens were discovered in the vast material designed to serve a large project at University of Würzburg by AF to study the ecology of arthropods living in tree canopies in tropical rainforest of Sabah, northern Borneo and assessing the effects of human disturbance in primary rainforest. In total, he fogged 334 tree canopies at a height of 5–20 meters (trees had an average height of 24 meters) in a variety of forest types including primary and secondary forests of different ages, altitudes and seasons, some adjacent to primary forest, others in isolated stands. Three major geographic areas were visited, ranging from 100–2000 m.a.s.l.,viz. the Kinabalu area, Crocker Range and Tawau Hills (Floren, partly unpublished data).

The majority of the spider material discussed here was obtained between 1992 and 2009 by fogging the lower canopy of 80 trees in Poring Hot Springs in the Mount Kinabalu area, in a mature primary Dipterocarp rainforest at approximately 700 m.a.s.l. Five tree species were focused upon: (*Xanthophyllum affine* [Polygalaceae], three species of *Aporosa* [Phyllanthaceae] and *Aglaia macrophyllum* [Meliaceae]). In 1997, 48 tree samples in secondary forests of different age, adjacent to primary forest were sampled in a lowland Kinabalu site north of Poring (Sorinsim). Here, 18 trees of *Mallotus* (Euphorbiaceae) and 15 *Vitex pinnata* trees (Verbenaceae) were fogged. All spiders were sorted to family and identified as morphospecies and where possible to species and analysed ecologically (Floren and Deeleman-Reinhold 2005). At the moment of writing, 749 morphospecies have been recognised and documented, partly described and named, most still unidentified or unidentifiable. Supplemental samples are still being analysed regularly. All material is deposited in Naturalis Biodiversity Center, Leiden (RMNH).

Drawings were made with a Zeiss Stemi SV 11 stereo dissecting microscope with drawing tube. Photographs were made with NIKON DS-R:1 driven by NIS Elements software and mounted on the M165 C stereomicroscope, using Auto-Montage software version 5.03 (JM), and a Fuji camera Finepix HS 20 with the Zeiss Stemi, (Fig. 5, CD-R). Measurements reported in millimeters.

## Taxonomy

### *Depreissia* Lessert, 1942

**Type species.** *Depreissia myrmex* Lessert, 1942 (♂)

#### Depreissia decipiens

Taxon classificationAnimaliaAraneaeSalticidae

Deeleman-Reinhold & Floren, 2003

Figs 1–4 , 5–10 , 11–13 , 14

Depreissia decipiens Deeleman-Reinhold & Floren 2003: 336, figs 1–7 (description ♂ holotype)

##### Holotype.

**MALAYSIA (BORNEO)**: Sabah, Kinabalu area, Sorinsim, ♂, 40 year old secondary forest adjacent to primary forest, 6°17'52"N, 116°42'3"E, 280 m.a.s.l., canopy fogging *Vitex pinnata* (L.), (Verbenaceae), tree 9 fog 1, 8.3.1997.

##### Additional material.

Sabah, 1 immature, same as holotype, canopy fogging *Vitex pinnata* (L.) tree 5, fog 1, 7.3.1997; Mt. Kinabalu National Park at Poring Hot Springs, primary forest, 6°2'37"N, 116°41'57"E, 500–700 m.a.s.l., 1♀, canopy fogging, *Aporosa maingayi* tree 6, fog 1, 28.3.1998; Mt. Kinabalu National Park at Poring Hot Springs, 6°2'42"N, 116°41'54"E, 1 immature, canopy fogging , *Aporosa lagenocarpa*, tree 8, fog 1, 29.3.1998; Mt. Kinabalu National Park at Poring Hot Springs, 6°2'58"N, 116°41'58"E 1 immature, *Aporosa lagenocarpa* tree, 19.2.1996; Kinabalu area, Monggis lowland primary forest, 6°13'17N, 116°44'14"E, 300 m.a.s.l., 2 immatures, canopy fogging *Lithocarpus* sp. (Fagaceae) tree 32, fog 1, 23.9.2006 (Fig. 14). All A. Floren, deposited in Naturalis Biodiversity Center, Leiden (RMNH).

##### Diagnosis.

The palpal structure is unlike that in any known salticid. *Depreissia decipiens* shares with the African species *Depreissia myrmex* the peculiar elongate, dorsally flat rhombus-shaped carapace, and the PLE widely separated and far removed from the anterior eyes. The rear part of the carapace is transformed. The palpal tegulum has a deep constriction, which divides it into an anterior and a posterior part, the latter bearing an elongate bifid tegular apophysis and a large chitinized grooved median apophysis directed downward along the longitudinal axis. The spermophore and filiform embolus are situated in the anterior part (Figs 12, 13).

*Depreissia decipiens* male is distinguished from the African species *Depreissia myrmex* by its larger size and accordingly longer legs, pale orange colour rather than dark as in *Depreissia myrmex*, and the ALE positioned posterolaterally instead of directly behind the AME. The posterior end of the carapace in *Depreissia myrmex* has an upturned posterior margin (Wesołowska 1997: fig. 2); in *Depreissia decipiens* there is a central pustulous hump and a pair of large lateral pits (Figs 5, 8, 9; Deeleman-Reinhold and Floren 2003: fig. 6). Cheliceral promargin with 2 teeth (one in *Depreissia myrmex*). Femora and some other segments of leg I with at least one spine (legs of *Depreissia myrmex* spineless). The pedicel is much longer and arched in *Depreissia decipiens*, shorter and straighter in *Depreissia myrmex*. Palp (Figs 11–13): the embolus runs transversely as half a coil along anterior half of the tegulum (Fig. 13), parallel to the spermophore, whereas there are two full coils in *Depreissia myrmex*. In *decipiens* the membranous tegulum is divided into an anterior and a posterior part, connected by a thin string of soft tissue, the posterior part bears at its base a bifid tegular apophysis (bta in Figs 11, 13) and a strong grooved dagger-like median apophysis which is directed basally (bta and ma, Fig. 13; Deeleman-Reinhold and Floren 2003: fig. 3); in *Depreissia myrmex* the apophysis is spout-like (Wesołowska 1997: figs 3–5).

**Figures 1–4.**
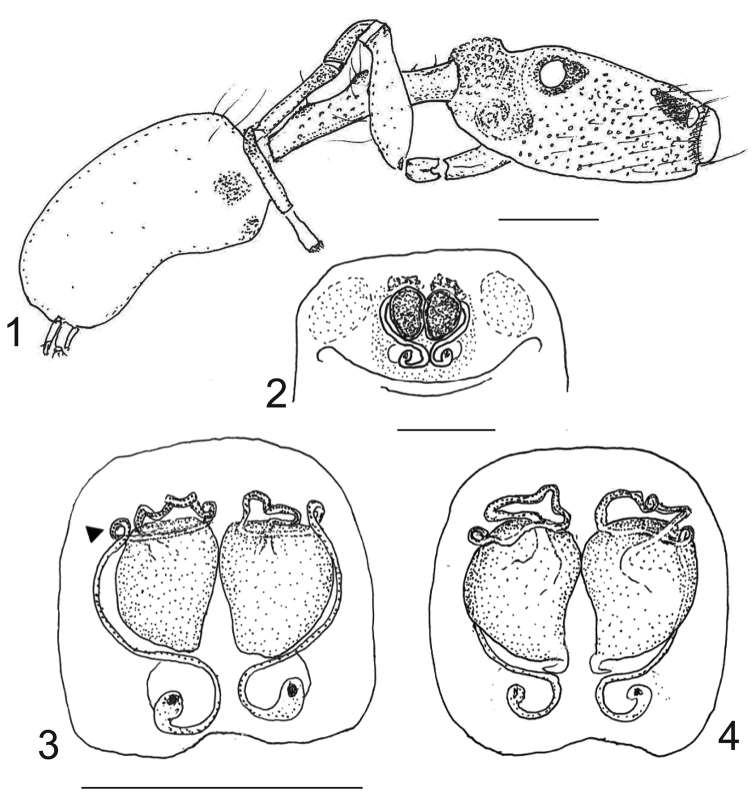
*Depreissia decipiens* female **1** Female habitus, lateral **2** epigyne in situ, ventral view **3** epigyne, ventral view, arrow indicates first tight loop **4** vulva, dorsal view. Scale bars: 0.4 mm (**1**), 0.2 mm (**2–4**).

**Figures 5–10.**
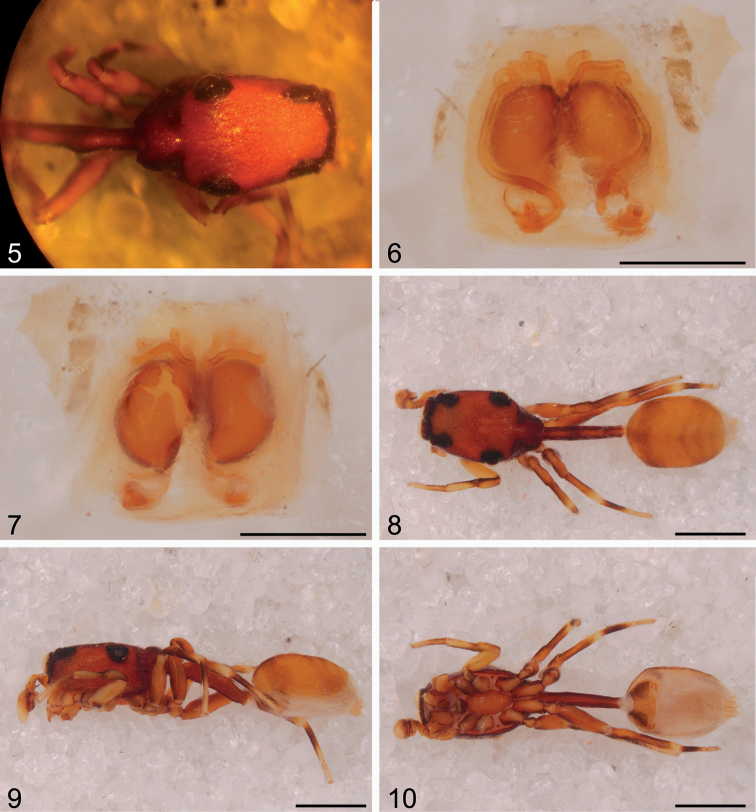
*Depreissia decipiens* **5** Female carapace and pedicel, dorsal view **6** epigyne, ventral view **7** vulva, dorsal view **8** male habitus, dorsal view **9** male habitus, lateral view **10** male habitus, ventral view. Scale bars: 0.2 mm (**6–7**), 1.0 mm (**8–10**).

**Figures 11–13.**
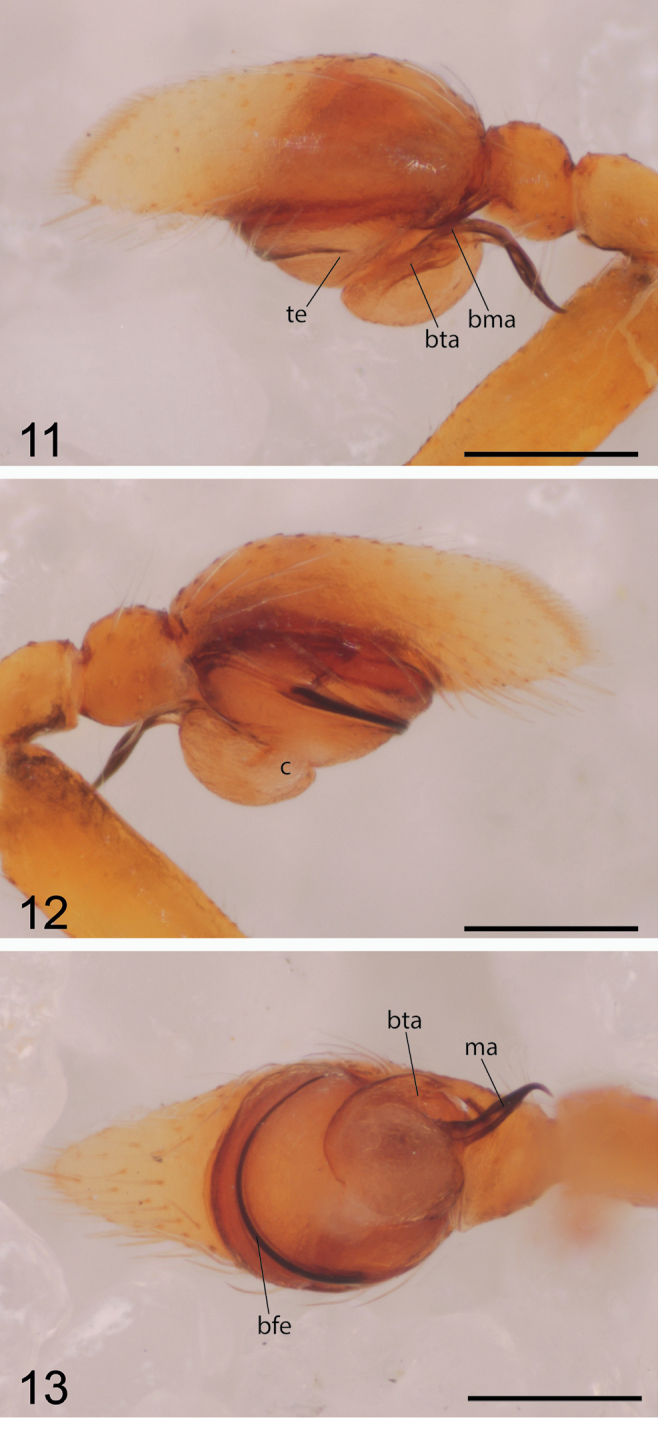
*Depreissia decipiens*, male palp **11** retrolateral view **te** tip embolus **bta** bifid tegular apophysis **bma** base median apophysis **12** prolateral view **c** membranous connection dorsal and ventral tegulum **13** ventral view **bfe** base free part of embolus **bta** bifid tegular apophysis **ma** median apophysis. Scale bars: 0.2 mm.

**Figure 14.**
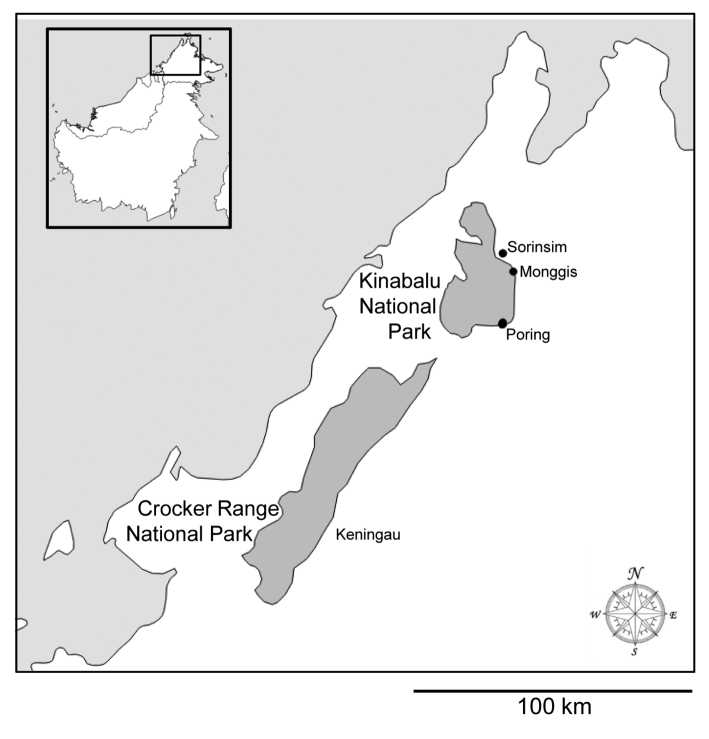
Specimen records of *Depreissia decipiens* (black circles) from the vicinity of Sorinsim, Monggis, and Poring Hot Spring. The locations of Kinabalu and Crocker Range National Parks, and the Keningau area, are shown. Inset shows area of map within Borneo.

##### Redescription of the male.

Total length 4.10. Carapace length 1.55, width 1.00, height 0.80. clypeus height 0.10, maxillar height 0.35, width 0.15 at base, 0.25 in the middle, 0.20 distally, chelicerae length 0.50, width 0.25 in the middle, pedicel length 1.25, abdomen length 1.50, width 1.00. Eye sizes: AME 0.30, ALE 0.11, PME 0.03, PLE 0.18. Eye row widths: AME-AME 0.60, ALE-ALE 0.72, PME-PME 0.75, PLE-PLE 0.95.

Leg I (coxa-trochanter-femur-patella-tibia-metatarsus-tarsus = total) 0.25-0.15-0.60-0.30-0.50-0.40-0.20 = 2.40, leg II 0.30-0.10-0.60-0.30-0.50-0.40-0.30 = 2.50, leg III 0.30-0.10-0.70-0.30-0.50-0.50-0.25 = 2.65, leg IV 0.45-0.20-0.90-0.45-0.55-0.50-0.30 = 3.35. Total length of right and left legs differ by approximately 3–4%.

Spider orange, carapace as in diagnosis, maxillae hooked outward at right angle, chelicerae basal half deeply excavated mesally (Deeleman-Reinhold and Floren 2003: fig. 5), promargin with 2 teeth, posterior margin with some granule-based setae. Intercoxal ventral triangles laterally between coxae II and III. Pedicel slightly shorter than abdomen, arched, with two prominent humps dorsally, surface dotted with seta-bearing granules. Legs vaguely banded orange/cream/black; all leg femora have one disto-dorsal spine; there are two lateral unpaired short thick spines proventrally on tibia I and one similar on metatarsus I; all other segments are spineless. Abdomen oblate with dorsal scutum over 5/6 of the dorsal surface, showing a vague broad dark band anteriorly, continued on the flank and one near the rear and with several chevrons in between. Pulmonary plates large, triangular.

Palp: see diagnosis. Tibial apophysis short and straight (Fig. 11). Cymbium tip lacking central groove, there is a clear rim bordering anteriorly the cavity which hosts the bulb. The embolus is directed retrolaterally, it is not cradled.

##### Description of the female

(Figs 2–7). Total length 4.40, carapace length 1.60, width 1.00, height 0.80, clypeus height 0.10, maxillar height 0.30, width 0.15 at base, 0.25 in the middle; chelicerae slightly excavated, 0.40 long, 0.20 in the middle, pedicel length 1.25, abdomen length 1.70, width 1.00. Legs I and II lost, leg III 0.40-0.20-0.70-0.30-0.60-0.50-0.30 = 3.00, leg IV: 0.50-0.25-0.90-0.40-0.55-0.50-0.30 = 3.40; palp femur length 0.40, width 0.10, tibia length 0.15, patella length 0.15, tarsus length 0.30, width 0.20.

Somatic characters in female similar to male, slightly larger with slightly longer legs. Eyes, sculpture of carapace, colour and integument ornamentation as in male; maxillae as in male, chelicerae simple, mesal excavation only slight, with two small teeth. Palpal tarsus white, slightly flattened, no claw visible with microscope objective lens 6.6 x enlargement. Intercoxal ventral triangles and pedicel as in male. Femoral spines weak or absent. Abdomen lacking dorsal scutum.

Epigyne: spermathecae relatively large, adjacent to one another in the vertical midline. Copulatory openings posterior, funnel-shaped, with a small atrium and continued in a narrow tubiform duct (insemination duct), almost straight, bordering the spermathecae along lateral-ventral sides. The duct tightly loops mesally (see arrow in Fig. 3), to continue along the dorsal surface of the spermathecae, then returns back lateral-wards in a series of vertical coils; at the lateral end it returns back straight on the ventral side again to dive down at mesal end to merge into the spermathecae.

##### Note.

In many non-salticid spider species with a filiform embolus have it supported during copulation by a sclerite. We suppose that in this salticid the conspicuous s-shaped dagger-like median apophysis ventrally on the tegulum of the male palp serves that purpose. It penetrates the opening of the insemination duct supporting the embolus, the free part of which it matches in length (Fig. 13); this length approximates also the length of the spermathecae, so that the tip of the embolus and median apophysis reach approximately the point of the first tight loop (arrow, Fig. 3). The sperm consequently has to travel on towards the spermathecae through the long trajectory of twists and curls of the insemination duct. It can be expected that the inner wall of the distalmost section is clothed with specialized cells or glands.

##### Relationships.

It is difficult to assess the taxonomic position of *Depreissia* within the Salticidae. There is complete lack of conformity in body shape and structure of genital organs with the ant-mimicking Myrmarachninae. Neither do they fit in Lyssomaninae: eyes in 4 rows instead of 3 characterizes *Depreissia myrmex* but not *Depreissia decipiens* and the genital organs are of a clearly different type. The structure of both palp and epigyne in *Onomastus* Simon has a certain superficial resemblance (Benjamin 2010, Prószyński and Deeleman-Reinhold 2013), but the conformation of genital organs is incompatible with that in *Depreissia*. The bipartite tegulum, the embolus tip not resting on cymbium tip, the presence of a sophisticated median apophysis and the unusual structure of the epigyne are key factors. A median apophysis is found mostly in primitive salticid clades, such as Cocalodinae (Maddison 2009). In *Depreissia*, the median apophysis is positioned on the tegulum clockwise and at some distance from the embolus. The nearest genera with a comparably structured palp with similarly positioned median apophysis can be found in a recently described series of genera from New Guinea (Maddison 2009). We suggest the species of the genus *Cucudeta* Maddison to be at present the nearest known relative of *Depreissia*. This is also supported by the similar structure of the epigyne, with openings in posterior pockets, long ducts rising forwards in lateral arch, looping, and entering the anterior end of the spermathecae (see also Maddison et al. 2016). Although morphology in *Cucudeta* is not particularly ant like, this spider has been observed walking with a specific fluid gait while keeping the second pair of legs in the air (Maddison 2009), suggesting a tendency toward ant mimicry.

## Which model is *Depreissia* mimicking?

**Figures 15–16.**
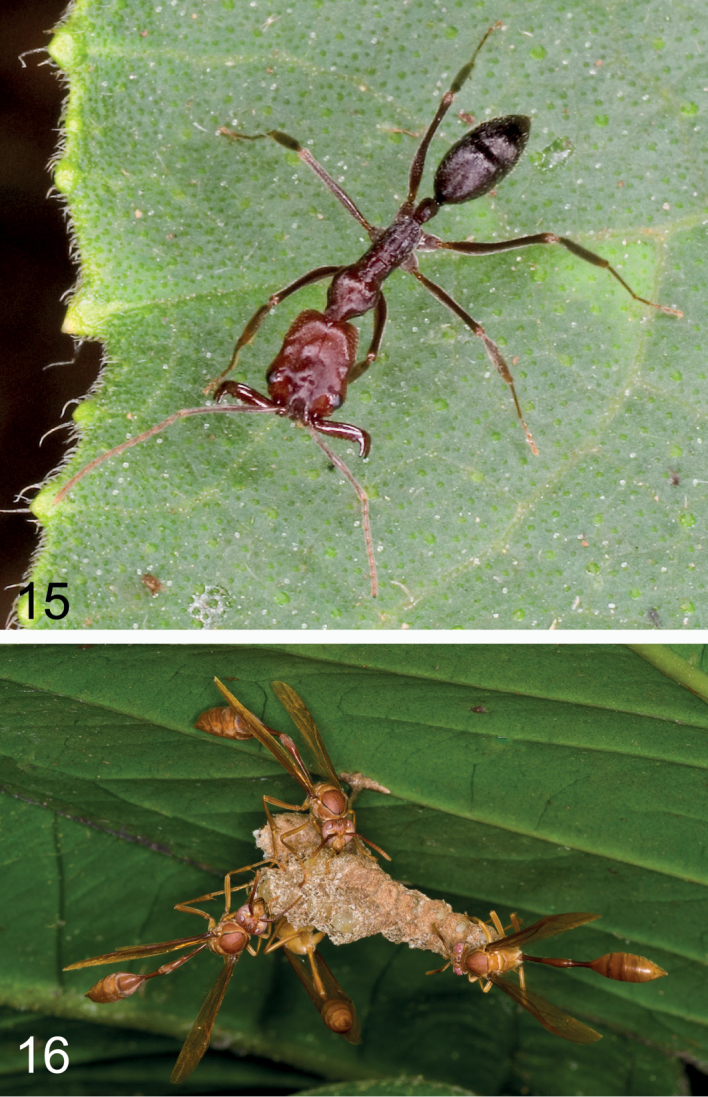
Possible hymenopteran models for *Depreissia decipiens* mimicry **15** snap jaw ant (*Odontomachus*) **16** social wasps (Polistinae). Photo credits Paul Zborowski (close-up-photolibrary.com).

The most common ant species in the canopy stratum in the Poring area belong to the genera *Camponotus* Mayr, *Polyrhachis* Smith, *Dolichoderus* Lund and *Crematogaster* Lund (Floren et al. 2001, 2002). According to general morphology, these ants can be considered to be the model to any of the numerous ant-mimicking species of *Myrmarachne* MacLeay, *Bocus* Peckham & Peckham and *Agorius* Thorell (Salticidae) and two species of *Corinnomma* Karsch (Corinnidae) found in the canopy of the same trees. It is hard to imagine that any of these ants serves as model for *Depreissia* as well. The general habitus is completely different from that in the mentioned ant-mimicking genera. The division of the cephalothorax into distinct head and thorax regions is prominent in typical ant-mimicking salticids and corinnids; the cephalothorax of *Depreissia* lacks any such division. Characteristic of *Depreissia decipiens* is the very long pedicel. Some snap-jaw ants of the ponarine genus *Odontomachus* Latreille (Fig. 15) have a habitus that could be compared to *Depreissia*, but ants of that genus are barely present in the canopy at Poring (Brühl et al. 1998: fig. 1, p. 289). Ants with long waist are supposed to be mimicked by several *Myrmarachne* species with long pedicel present in the canopy.

Some spiders of the family Corinnidae (*Aetius* O. Pickard-Cambridge, *Coenoptychus* Simon) are known to mimic other hymenopterans, such as mutillid wasps (Karsch 1892, Majumder and Tikader 1991). The elongate head borne horizontally with a conspicuous gap with the pronotum distinguishes all kind of ants from wasps; the latter rather have smaller heads tucked in vertical position underneath the pronotum, producing a better imitation of a spider cephalothorax.

Wasps with long thin pedicel are found in several subfamilies of Vespidae, such as Stenogastrinae and Polistinae. Two genera of paper wasps (Fig. 16), *Belonogaster* Saussure and *Ropalida* Guérin-Méneville, have a habitus similar to *Depreissia*. These genera are widespread in tropical Africa, Madagascar and tropical Asia. Similar wasps have been frequently observed by us in Sabah forests. They have not yet been analysed taxonomically. They are known to build colonial nests among leafy branches.

In mimicry complexes, behaviour of the mimic often reinforces and enhances the efficacy of morphological adaptation. So some clues to the mimicry model may be manifested only as activity of the live animal. For example, mimicry may be actively enhanced by emitting scent, airborne vibrations, or behaviour such as gait and movement of the first legs. Ant-mimicking spiders often hold the first or second leg-pair lifted and stretched forward to mimic antennae. The castianeirine spider *Pranburia mahannopi* Deeleman-Reinhold, 1992 is unique as an ant-mimic, having thick brushes on the first femora; only when disturbed, it joins its first legs in front of the head, creating a quite convincing ant’s head with antennae (Deeleman-Reinhold 1993). Behaviour in *Depreissia* remains completely undocumented. It may well be that no human has ever observed one alive.

**Distribution and species richness.** The existence of two closely related species, sharing an array of unusual characters not seen in other salticids and living isolated on two different continents and habitats is difficult to understand. Both species are extremely rare. *Depreissia decipiens* is known only from two adult specimens from 128 fogged trees in a limited area, (Fig. 14), situated within a biodiversity hotspot (Mittermeier et al. 1998). Mt. Kinabalu is an easily accessible location that has been relatively well surveyed, but no specimens have been found from lower forest strata. Why is it so rare?

The genus *Depreissia* might be considered to be relict of an ancient fauna that has vanished from the large area in between. This is not supported by the estimated young age of the Kinabalu Mountain of 6 million years (Cottam et al. 2013, Merckx et al. 2015). Another possibility could be that speciation occurred after long-distance dispersal. A third explanation is that *Depreissia* is widespread, only the species is rarely found because of low abundance, cryptic life style or living in inaccessible niches within the canopy which would obscure them from the scientific world.

*Depreissia decipiens* was only found in the canopy. Searching through thousands of photographs posted online depicting *Myrmarachne* and other interesting ant-mimicking spiders and insects, we did not find any capturing *Depreissia*. Photographers do occasionally document rare spiders, such as the corinnid *Pranburia* Deeleman-Reinhold. But *Depreissia* remains unseen for both photographers and scientists. Does *Depreissia decipiens* occur beyond the Mt. Kinabalu region? Are there still undiscovered *Depreissia* species in Asia? We shall have no answers without more arthropod surveys in tropical Asia that include sampling in primary forest canopy.

During a sampling course for students in a forest in Congo (Jocqué et al. 2013: 20), six collecting methods were compared. Canopy fogging was found to be the most productive of these sampling methods. This is also our experience: the fogging method produces larger amounts of material with longer series per species and fewer singletons than in hand-collected material.

Is *Depreissia* part of a fauna which is restricted to the canopy? In Sabah, fogging has provided a large volume of material from the canopy. Unfortunately comparing canopy fauna with that living in the lowest stratum is biased by lack of comparable sample data. Nevertheless, a few anecdotes are available to illustrate consistent differences between canopy spider community and that of spiders living in the understorey. The most convincing example of canopy-restricted spiders is provided by one very successful species of crab spider belonging to an unknown genus. In Poring Hot Springs this was one of the most dominant spiders in the canopy, it kept popping out in almost every tree sample in the primary forest in Kinabalu (but in none of the secondary forests sampled in Sabah), with a maximum of 300 individuals in 11 trees fogged in 1998, (the memorable year that Borneo and Sumatra were on fire). Most remarkable therefore is the single specimen collected by hand of this species in the primary forest at Danum Valley Field Centre, 160 km to the east (CD-R) in the crown of a freshly logged tree.

Certainly one of the best-studied spider genera for Borneo is *Myrmarachne*. Nearly 100 species have been recorded from Southeast Asia (World Spider Catalog 2015). Collectors including Takeshi Yamasaki, Peter Koomen, and others have hunted frequently in the Kinabalu region, focussing on *Myrmarachne*. All told, we recognize 29 species from lower strata in Sabah forests, nearly all from old primary and adjacent secondary forest in the Kinabalu area (Yamasaki and Ahmad 2013, Yamasaki 2010, 2012, Peter Koomen, personal communication). In our canopy samples, we distinguish 23 *Myrmarachne* species, 12 of which have also been found in lower strata.

There are indications from genital anatomy that 11 (43%) of the *Myrmarachne* species associated with the canopy in the Mt. Kinabalu area are taxonomically distinct and should be transferred to a new genus (Prószyński 2015a, 2015b). Of these, 5 are undescribed, often closely related to known species from the ground and are so far known from the canopy only.

Habitat preferences in what may be thought of as true *Myrmarachne* seems to contrast with those of prospective related genera, with true *Myrmarachne* species dominating lower forest strata and the prospective new genera dominating the canopy in primary rainforest in Poring and the Kinabalu mountain forest at 1500–2000 m a.s.l. In the secondary forest canopy in Kinabalu area (Sorinsim) the opposite is true and *Myrmarachne* species are dominant. This tendency may be determined by their models, the ants. Ant research carried out in Poring has found a clear stratification among ant tribes: myrmicines and ponerines dominate the forest floor, whereas formicines are most frequent in the canopy (Brühl et al. 1998: p.289, fig. 1). Any conclusion that distribution of myrmarachnine genera is reflected in that of the mimicked models may be unjustified, as key characters distinguishing myrmarachnine genera are mainly in genital organs, much less in somatic appearance.

## How many spider species live in tree canopies in Sabah?

When assessing the identity of canopy spiders in tropical rainforests of Borneo, evidence turned up again and again that not only on the forest floor, but also in the canopy, species ranges are often small and endemism appears to be high; more endemism means higher regional species richness. Experiments in the Sabah canopy project (Floren et al. 2011) were involved with investigating effects of isolation in separated patches of forest. It became clear that a large part of primary forest spider species have a restricted dispersal ability, less than 10 km.

The canopy at the fogging sites in our project has an average height of 24 meter; this means that the volume of the canopy habitat is ten times that of the 2.5 meter understorey, in which traditionally most collecting and inventories of fauna are carried out. It is not surprising that the number of foliage-associated species collected by fogging is very much larger than in ground collecting. Furthermore, the physical conditions of life on shrub and small trees growing on the forest floor in the shadow of big trees are quite different and the species composition differs basically from that higher up towards the crown. The canopy conditions are somewhat similar to that in younger secondary forest. The species richness in the latter is very variable: when influx from adjacent old primary forest is possible it may be very high such as in the older forest at Sorinsim; in isolated secondary forest (Keningau) the species community is much poorer and dominated by versatile widespread species.

With 2/3 of the Sabah canopy material processed, we distinguish 749 morphospecies in 36 families. 173 of these species till now have been assigned to known species, some widespread or even cosmopolitan. Most rich in species are Theridiidae (177), Salticidae (144), Thomisidae (98) and Araneidae (87). Corinnidae, Salticidae, Oxyopidae, Theridiidae and Tetragnathidae are the most versatile and include the highest percentage of widespread species (Floren and Deeleman-Reinhold 2005).

Within the realm of spider taxonomy, Salticidae are an intensively studied group, especially in species-rich areas in the world’s tropics (Prószyński 2012, 2015a, 2015b, Prószyński and Caleb 2015). Approximately 710 species in 160 genera are listed for Southeast Asia (excluding China and Pacific) (World Spider Catalog 2015); 127 species are specifically listed for Borneo. In the Sabah project, at present, 144 morphospecies of salticids are recognized in the canopy; as far as possible, of these, 28 species have been identified as described or otherwise registered species from the lower strata.

As we continue processing through the later material, species numbers rise steadily. We would like to mention that we examined two recently recovered additional tree samples from Poring from an under-collected season: one from an *Aporosa* tree contained 11 salticid species, three of which proved to be different from all previously registered species from the whole project. The other sample was from an *Aglaia* tree (Meliaceae) which contained 7 salticid species, two of which were new for the project. There was no overlap between the two trees.

In Thomisidae, 98 morphospecies are listed, including 19 known species, most recorded from various parts in S and SE Asia, sometimes widespread, many described by the end of the 19^th^ century and not or barely mentioned afterwards. For example, one single, quite distinctive species (*Thomisus perspicillatus* Thorell, 1890) from Sarawak has not been cited since its discovery.

In Clubionidae, 30 species were found in the canopy, only three were found in a wider area, up to Sumatra and probably the Philippines. The number shared with the ricelands in the Philippines (Barrion and Litsinger 1995) is difficult to assess: many species were described, most from one sex only, and with drawings showing minor differences in genitalia. It is worth mentioning here that CD-R examined the Clubionidae from a canopy fogging project in Papua New Guinea (spiders kept in KBIN, Brussels). 84 trees in a one km^2^ area in Baitete forest were fogged in 1993–1995, containing 38 clubionid species (maximum of 13 species in one tree) in a more diverse array of genera than in Sabah. Only one widespread species (*Pteroneta saltans* Deeleman-Reinhold, 2001) proved to be in common with the canopy fauna in Sabah (Versteirt et al. 2008, Versteirt et al. 2010). In Castianeirinae, 13 morphospecies have been identified, seven of which are widespread in SE Asia. Tetragnathidae are represented with 17 species, nine of which are well-known and widespread.

According to the Spider Catalog, 155 species of Theridiidae have been recorded for the Malay region, 14 for Borneo (World Spider Catalog 2015). In Kinabalu and Keningau (isolated disturbed lowland forest near Crocker Range) alone we recorded 177 species. Just some examples of genera that we have studied recently: *Molione* Thorell was one of the many dominant genera in the canopy with several hundreds of specimens in nine species. Two species (*Molione christae* Yoshida, 2003 and *Molione kinabalu* Yoshida, 2003) were found only in their type locality in primary and adjacent secondary forest respectively. *Molione uniacantha* Wunderlich, 1995 is widespread in Malaysia and Borneo; in the canopy it was found only at one site in the Crocker Range, where it was abundant. A species resembling *Molione lemboda* Gao & Li, 2010 was found in a fruit plantation in Crocker Range. Most of 5 remaining species were singletons or rare.

The theridiid genus *Borneoridion* Deeleman and Wunderlich brought a surprise. This genus belongs to an aberrant lineage within the Theridiidae and was described recently for a single species found in the canopy of Keningau (Crocker Range lowland) (Deeleman and Wunderlich 2011). In this genus we found a consistent tendency to endemism. In four different forest sites we encountered six more new species, each with clearly distinct genitals in an array of similar somatic characters. No specimens of this genus have at present been found in the understorey.

When scrutinizing the various families in the project, several more poorly known genera emerged that show strong tendency to geographic speciation. One of them is the genus *Gephyrota* (Philodromidae). In this genus, 4 species are known from South and Southeast Asia (two species from juveniles only), all described by Simon before 1910; no records have been published since (World Spider Catalog 2015). In Borneo, we found five new species in the canopy in five different forests, each locality with its own species.

In Araneidae, we found a group of approximately five distinct species (30 adult specimens), possibly near *Chorizopes* O. Pickard-Cambridge, characterized as small (<3 mm) spiders with a cylindrical, strongly ridged warty abdomen that resembles a tree bud; we found them exclusively in the Kinabalu area and as far as we can ascertain are undocumented in literature.

These examples are representative of many similar cases that have emerged from years of study on the biodiversity of Southeast Asian spiders. High regional diversity is the product of high species richness within locations combined with short-range endemism of many of those species. One lesson from this investigation into the tropical rainforest arthropod community comes through incontrovertibly: the number and variety of life forms, and of their interactions in the tropical forest canopy seems to have no end, and we still know only a fraction so far. Decades after the introduction of fogging as a method for accessing the forest canopy fauna (Erwin 1983), this community remains a scarcely explored frontier of discovery.

## Supplementary Material

XML Treatment for Depreissia decipiens
